# Outcomes of durable versus biodegradable polymer drug-eluting stents in patients with coronary artery disease

**DOI:** 10.1016/j.ijcha.2026.101882

**Published:** 2026-02-06

**Authors:** Christof Skos, Jovan Rogozarski, Al Medina Dizdarevic, Gloria M.Steiner-Gager, Marek Postula, Ceren Eyileten, Aurel Toma, Walter S. Speidl, Nika Skoro-Sajer, Christian Gerges, Irene M. Lang, Jolanta M. Siller-Matula

**Affiliations:** a Department of Medicine II, Division of Cardiology, Medical University of Vienna, Vienna, Austria; b Department of Experimental and Clinical Pharmacology, Center for Preclinical Research and Technology CEPT, Medical University of Warsaw, Poland

**Keywords:** Percutaneous coronary intervention, Coronary artery disease, Acute coronary syndrome, Chronic coronary syndrome, Drug-eluting stents, Target lesion revascularization

## Abstract

**Background:**

Percutaneous coronary intervention (PCI) is among the most common cardiovascular procedures, but stents still pose risks of restenosis or thrombosis. Drug-eluting stents (DES) with polymer coatings have improved long-term outcomes.

**Aim:**

This study evaluated whether the latest biodegradable polymer DES (BP-DES) offer improved safety over durable polymer DES (DP-DES).

**Methods:**

Data were collected from a single-centre registry at the Medical University of Vienna, including patients who underwent PCI between 2015 and 2020. Patients were categorized by stent type and PCI indication (All Comer, CCS-PCI, ACS-PCI). The primary endpoint comprised of major adverse cardiac events (MACE), including target lesion revascularization (TLR), target vessel revascularization (TVR), stent thrombosis (ST), and all-cause death.

**Results:**

2118 patients were eligible for further analysis. 1232 patients (58.2%) received a DP-DES. In the all-comer cohort, 5-year MACE rates were 12% for BP-DES vs 14.5% for DP-DES. Multivariate analysis showed no significant difference in MACE for the use of BP-DES (OR 0.941, 95% CI 0.734–1.207, p = 0.631). However, TLR rates were significantly lower in patients treated with BP-DES (3.4% vs 6.8%, OR 0.567, 95% CI 0.372–0.865, p = 0.008), mainly driven by lower rates of TLR within the ACS PCI cohort (1.5% vs 4.9%, OR 0.364, 95% CI 0.158–0.841, p = 0.018). In CCS PCI patients, MACE and TLR rates demonstrated no significant differences.

**Conclusion:**

BP-DES and DP-DES show a similar long-term safety profile regarding MACE. BP-DES demonstrate a lower risk of TLR in the all-comer cohort, driven by reduced rates in ACS-PCI patients.

## 1 Introduction

Improvement of current drug-eluting stent (DES) technology is an ongoing process. Unlike durable polymer drug-eluting stents (DP-DES), biodegradable polymer drug-eluting stents (BP-DES) are designed to leave only the thin, endothelialized metal struts within the stented region. The absence of a permanent polymer may contribute to reducing chronic inflammatory processes, due to the limited duration of polymer presence within the stented region compared to DP-DES. After implantation, the polymer of BP-DES remains for approximately 4–12 months, depending on the stent platform, compared to a lifelong persistence of a DP-DES polymer, which has been associated with chronic vascular inflammation [1], [2], [3].

Whether BP-DES outperform DP-DES in terms of long-term patient outcomes remains a topic of continuing discussion, although overall differences appear to be marginal [4], [5], [6].

## 2 Materials and Methods

### 2.1 Aim of this study

**Fig. 1:**
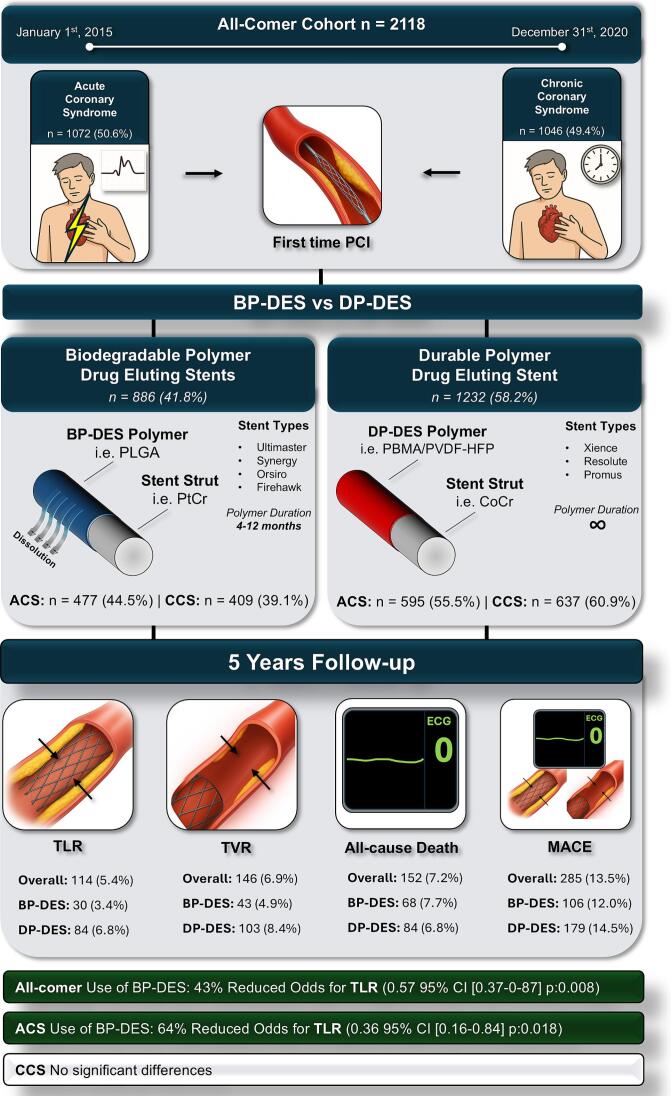
Central Illustration.

The main objective of this registry study was to determine whether BP-DES outperform DP-DES regarding major adverse cardiac events (MACE) in a real-world setting. Patient outcomes were analysed up to 5 years after index PCI. Subgroup comparisons between BP-DES and DP-DES were conducted for an all-comer cohort, an ACS-PCI cohort, and a CCS-PCI cohort (Fig. 1).

### 2.2 Study design

**Fig. 2:**
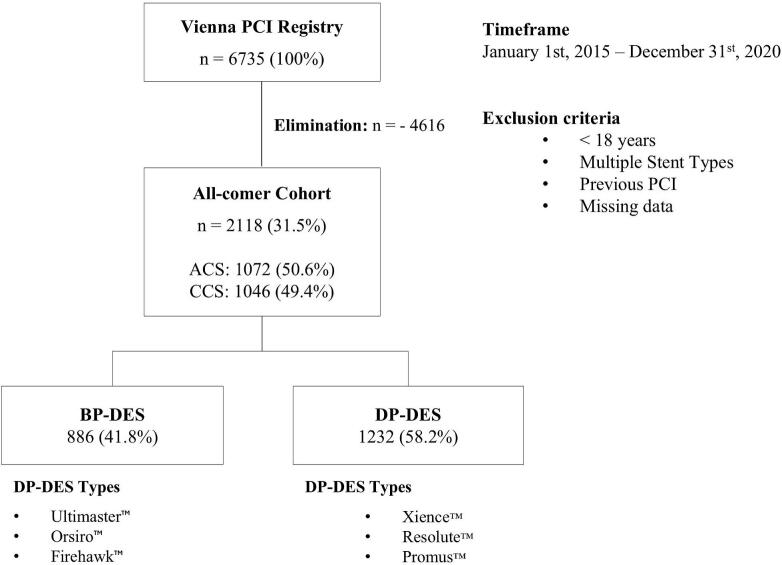
Study Design Flow Chart.

This study was conducted as a cohort study, with data collected from the Cardiac Catheterization Registry of the Medical University of Vienna, which has been described previously [7]. All patients aged 18 years or older who received either a BP-DES or DP-DES according to current hospital practice between January 1, 2015, and December 31, 2020, were included (Fig. 2).

The DP-DES types included the models Xience™ DES (Abbott Cardiovascular, Plymouth, MN, USA). Resolute™ DES (Medtronic, Dublin, IRL) and Promus™ DES (Boston Scientific, Marlborough, MA, USA), the BP-DES Types included were Ultimaster™ DES (Terumo, Shibuya, JPN), Synergy™ DES (Boston Scientific, Marlborough, MA, USA), Orsiro™ DES (Biotronik, Berlin, GER) and Firehawk™ DES (MicroPort, Shanghai, CHN). A comprehensive table summarizing the technological stent properties of each stent used within this study is provided in Table 1.

**Table 1:** Properties of drug eluting stents from this study.

	**Xience**	**Resolute**	**Promus**	**Ultimaster**	**Synergy**	**Orsiro**	**Firehawk**
Manufacturer	Abbott	Medtronic	Boston Scientific	Terumo	Boston Scientific	Biotronik	Microport Medical
Polymer Type	Durable Polymer	Durable Polymer	Durable Polymer	Biodegradable Polymer	Biodegradable Polymer	Biodegradable Polymer	Biodegradable Polymer
Polymer Material	PBMA/ PVDF-HFP	PBMA/PHMA PVP/PVA	PBMA/ PVDF-HFP	PDLLA-PCL	PLGA	PLLA	D,L-PLA
Polymer Thickness, μm	8	6	8	15	4	7	10
Polymer Duration, months	∞	∞	∞	4	4	12	9
Anti-proliferative Agent	Everolimus	Zotarolimus	Everolimus	Sirolimus	Everolimus	Sirolimus	Sirolimus
Coating Type	Circum-ferential	Circum-ferential	Circum-ferential	Abluminal	Abluminal	Circum-ferential	Abluminal
Strut Material	CoCr	CoCrPtIr	PtCr	CoCr	PtCr	CoCr	CoCr
Strut Thickness, μm	81	81	81	80	74	60	96

PBMA: poly n-butyl methacrylate; PVDF-HFP: poly-vinylidene-co-hexafluoropropylene; PDLLA-PCL: poly-D, L-lactic acid; PLGA: poly-lactic co-glycolic acid; PLLA: poly-L-lactic acid; D,L-PLA: D,L-poly-lactic-acid; CoCr: cobalt chromium; PtIr: platinum iridium; PtCr: platinum chromium, Strut thickness at 3,5mm stent diameter.

Source: [37], [38].

Patients were not eligible for this study, if multiple stent polymers were used during the key intervention, if a history of previous PCI was obtained or if follow-up data were inconclusive due to missing information. This trial was conducted in accordance with the Declaration of Helsinki and approved from the Ethics Committee of the Medical University of Vienna (1487/2021).

### 2.3 Data collection

Data collection and follow-up investigation took place from June 1, 2021, to February 10, 2023. All relevant coronary intervention datasets were extracted from the clinical department of cardiology interventional database. Mortality data was obtained from the Austrian Federal Statistical Office (Vienna, AUT). Patient characteristics were retrieved from the hospital’s electronic health record system. The trial database was locked on November 10, 2023. Statistical analysis was conducted using SPSS 30® (IBM, Armonk, NY, USA) and R (Version 4.4.1., Foundation for Statistical Computing, Vienna, Austria).

### 2.4 Primary and secondary endpoints

The primary endpoint was a composite endpoint of major adverse cardiac events (MACE), comprising clinically driven target lesion revascularization (TLR), target vessel revascularization (TVR), definite stent thrombosis (ST) and all cause death. Clinically driven target lesion revascularization was defined as any repeat revascularization at the treated segment including the 5-mm margin proximal and distal to the stent [8]. Clinically driven target vessel revascularization was defined as any repeat percutaneous intervention of any segment of the target vessel including the target lesion [8]. Definite stent thrombosis was defined according to the Academic Research Consortium [8]. The secondary endpoints of this study were the individual components of the primary endpoint.

### 2.5 Statistical analysis

A broad statistical approach was used to compare groups and perform time to event analyses. Statistical significance was set at a two-sided p-value of less than 0.05. Results are presented as odds ratios (OR) with 95% confidence intervals (95% CI). Continuous data were expressed as medians with interquartile ranges (IQR) and compared using the Mann-Whitney *U* test. Categorical variables were compared using the chi-square test and presented as numbers with proportions. The Kaplan-Meier method and log-rank-test were used for time to event analysis with up to 5-years follow-up time available for the primary and secondary endpoints.

To assess whether the use of BP-DES was an independent predictor of favourable outcome on primary and secondary endpoint measures at the 5-year follow-up, a multivariate Cox regression analysis was conducted containing the following variables: age, sex, obesity, diabetes mellitus, estimated glomerular filtration rate (eGFR) and C-reactive protein (CRP).

**Fig. 3:**
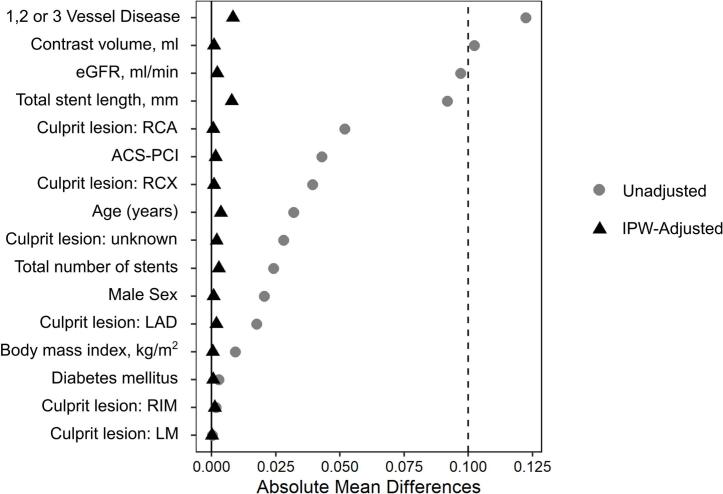
Covariate Balance before and after inverse probability weighting (IPW).

To address potential selection bias due to non-randomized stent allocation, a confirmatory inverse probability weighted analysis (IPW) based on propensity scores (PS) was applied. Propensity scores were estimated based on variables relevant to stent choice. Covariate balance after IPW was assessed using standardized mean differences (SMD), with values below 0.1 considered indicative of adequate balance. The variables included in the calculation of the PS are listed in Fig. 3.

## 3 Results

### 3.1 All-comer PCI cohort

In our study, 2118 patients undergoing PCI between January 1, 2015, and December 31, 2020, were eligible for further analysis. The baseline characteristics are presented in Table 2. Median follow-up duration was 223 days (IQR: 105–377) in the DP-DES group and 202 days (IQR: 76–282) in the BP-DES group. The median patient age was 66 years (IQR: 56–76) and 1553 patient (73.3%) were male. 1903 patients (89.8%) with hypertension and 630 patients with diabetes mellitus (29.7%) were registered. The median body mass index (BMI) was 27.1 kg/m^2^ (IQR: 24.6–30.5).

**Table 2:** Baseline Characteristics All Comer Cohort.

	**All-comer cohort (n = 2118)**			
	**Overall *(n=2118)***	**BP-DES *(n=886)***	**DP-DES *(n=1232)***	***p-value***
Age (years)	66 (56-76)	65 (56-75)	66 (57-76)	0.290
Male Sex	1553 (73.3)	642 (72.5)	911 (73.9)	0.446

**Risk Factors / Comorbidities, n (%)**				
Body mass index, kg/m^2^	27.1 (24.6-30.5)	27.1 (24.6-30.25)	27.1 (24.6-30.7)	0.700
Obesity, n (%)	550 (26.0)	217 (24.5)	333 (27.0)	0.189
Hypertension	1903 (89.8)	797 (90.0)	1106 (89.8)	0.891
Dyslipidaemia Treatment	2118 (100.0)	886 (100.0)	1232 (100.0)	.
Diabetes mellitus	630 (29.7)	263 (29.7)	367 (29.8)	0.958

**Interventional Characteristics**				
Elective PCI	1046 (49.4)	409 (46.2)	637 (51.7)	**0.012**
STEMI	659 (31.1)	297 (33.5)	362 (29.4)	**0.042**
NSTEMI	413 (19.5)	180 (20.3)	233 (18.9)	0.421
Culprit lesion: LAD	1017 (48.0)	432 (48.8)	585 (47.5)	0.562
Culprit lesion: RCA	581 (27.4)	227 (25.6)	354 (28.7)	0.113
Culprit lesion: RCX	377 (17.8)	183 (20.7)	194 (15.7)	**0.004**
Culprit Lesion: LM	24 (1.1)	9 (1.0)	15 (1.2)	0.665
Total number of stents (IQR)	1.0 (1.0-2.0)	1,0 (1.0-2.0)	1,0 (1.0-2.0)	0.764
Total stent length, mm	27.0 (18.0-41.0)	28.0 (18.0-43.0)	26.0 (18.0-40.0)	**0.039**
Maximum diameter stent, mm	3.0 (3.0-4.0)	3.0 (3.0-4.0)	3.0 (3.0-4.0)	0.058
Fluoroscopy time, minutes	16 (10-26)	17 (10-26)	15.9 (10.0-25.6)	0.218
Contrast volume, ml	180 (130-250)	190 (132-250)	180 (130-240)	0.054
Dose Area Product (DAP), cGycm^2^	7328 (4328-12894)	6886 (4035-12330)	7661.5 (4395.9-13532)	**0.019**

**Laboratory Data**				
Troponin T, pg/ml	62 (22-309)	60 (21-280)	63 (22-322)	0.746
NTproBNP, pg/ml	405.3 (112.9-1521.0)	374.8 (107.5-14280.0)	436.3 (114.4-1 571.0)	0.197
CK, U/L	121 (74-265)	112 (71-245)	125 (77-278)	**0.016**
CK-MB, U/L	45.7 (22.1-115.5)	49.5 (23.6-124.5)	42.2 (21.4-107.1)	**0.012**
Haemoglobin, g/dl	13.9 (12.5-15.1)	13.9 (12.6-15.1)	13.9 (12.5-15.1)	0.974
HbA1c, % (mmol/mol)	5.7 (5.4-6.2)	5.7 (5.4-6.2)	5.7 (5.4-6.3)	0.068
eGFR, ml/min	78.4 (58.5-92.6)	79.9 (60.7-93.1)	77.4 (56.6-92.1)	**0.036**
LDL-cholesterol, mg/dl	92.5 (62.7-122.1)	94.0 (62.6-124.0)	90.8 (62.8-120.4)	0.547
Triglycerides, mg/dl	123 (85-177)	123 (88-179)	123 (84-176)	0.419
Leukocytes, /l	9.1 (7-11.8)	9.2 (7.1-11.8)	9.0 (7.0-11.8)	0.534
CRP, mg/dl	0.3 (0.2-0.9)	0.3 (0.1-0.9)	0.4 (0.2-0.9)	0.188

**Concomitant Medication**				
Aspirin	1887 (89.1)	799 (90.2)	1088 (88.3)	0.173
DOAC	218 (10.3)	98 (11.1)	120 (9.7)	0.324
P2Y inhibitor	1736 (82.0)	736 (83.1)	1000 (81.2)	0.262
Beta-blocker	1771 (83.6)	745 (84.1)	1026 (83.3)	0.621
Calcium channel blocker	433 (20.4)	168 (19.0)	265 (21.5)	0.151
ACE inhibitor	1202 (56.8)	528 (59.6)	674 (54.7)	**0.025**
ARB	656 (31.0)	253 (28.6)	403 (32.7)	**0.041**
Statin	2088 (98.6)	875 (98.8)	1213 (98.5)	0.564

DOAC: direct oral anticoagulant; NTproBNP: N-terminal pro b-type natriuretic peptide; LDL: low-density lipoprotein, HbA1c: glycated haemoglobin; eGFR: estimated glomerular filtration rate, CRP: c-reactive protein, PCI: percutaneous coronary intervention, DES: drug-eluting stent, CK: creatine kinase, Lp-PLA2: lipoprotein-associated phospholipase A2, ACE: angiotensin-converting enzyme inhibitor, ARB: angiotensin receptor blocker, DAP: dose area product, (N)IDDM: (non–)insulin dependent diabetes, (N)STEMI: (non–)ST-elevation myocardial infarction, LAD: left anterior descending artery, RCA: right coronary artery, RCX: ramus circumflex artery, LM: left main coronary artery.

Regarding the indication for PCI, 1046 (49.4%) patients underwent elective PCI for CCS, 659 had ST elevation myocardial infarction (STEMI, 31.1%) and 413 non-ST elevation myocardial infarction (NSTEMI, 19.5%). Treatment with BP-DES was performed in 886 cases (41.8%) and 1232 patients were treated with DP- DES (58.2%). DP-DES were consistently used more frequently than BP-DES throughout the entire study period. Compared with DP-DES patients, BP-DES patients more frequently underwent PCI in the CX artery (20.7% vs 15.7%), demonstrated a longer median total stent length (28.0 mm vs 26.0 mm), and had a lower median dose area product (6886 cGycm^2^ vs 7661.5 cGycm^2^). Baseline characteristics were broadly similar between the two treatment groups, with no major differences observed. The following IPW analysis resulted in a cohort of 2005 patients. Compared to the unweighted cohort, an improved balance in all variables was achieved (Fig. 3, Table S1).

### 3.2 Major adverse cardiac events

In total, the primary endpoint MACE was observed in 285 patients (13.5%) with a median follow-up time of 577 days (IQR: 285–965). Clinically driven TLR occurred in 114 patients (5.4%), clinically driven TVR in 146 patients (6.9%), stent thrombosis in 7 patients (0.3%) and all-cause death in 152 patients (7.2%). Primary and secondary cumulative clinical outcomes are listed in Table 3.

**Table 3:** Summary of clinical outcome measures during follow-up categorized by Indication and Stent-type.

	**All-comer cohort**			**Acute coronary syndrome**			**Chronic coronary syndrome**		
	**Overall (n=2118)**	**BP-DES *(n=886)***	**DP-DES *(n=1232)***	**Overall (n=1072)**	**BP-DES (n=477)**	**DP-DES (n=595)**	**Overall (n=1046)**	**BP-DES (n=637)**	**DP-DES (n=409)**
**Major Adverse Cardiac Events**									
*30 days MACE*	31 (1.5)	12 (1.4)	19 (1.5)	18 (1.7)	4 (0.8)	14 (2.4)	13 (1.2)	8 (2.0)	5 (0.8)
*1-year MACE*	178 (8.4)	68 (7.7)	110 (8.9)	61 (5.7)	21 (4.4)	40 (6.7)	117 (11.2)	47 (11.5)	70 (11.0)
*5-years MACE*	285 (13.5)	106 (12.0)	179 (14.5)	107 (9.9)	38 (8.0)	69 (11.6)	178 (17.0)	68 (16.6)	110 (17.3)

**Target Lesion Revascularization**									
*30 days TLR*	13 (0.6)	4 (0.5)	9 (0.7)	9 (0.8)	1 (0.2)	8 (1.3)	4 (0.4)	3 (0.7)	1 (0.2)
*1-year TLR*	88 (4.2)	25 (2.8)	63 (5.1)	26 (2.4)	5 (1.0)	21 (3.5)	62 (5.9)	20 (4.9)	42 (6.6)
*5-years TLR*	114 (5.4)	30 (3.4)	84 (6.8)	36 (3.4)	7 (1.5)	29 (4.9)	78 (7.5)	23 (5.6)	55 (8.6)

**Target Vessel Revascularization**									
*30 days TVR*	20 (0.9)	6 (0.7)	14 (1.1)	13 (1.2)	2 (0.4)	11 (1.8)	7 (0.6)	4 (1.0)	3 (0.5)
*1-year TVR*	109 (5.1)	35 (4.0)	74 (6.0)	34 (3.2)	9 (1.9)	25 (4.2)	75 (7.2)	26 (6.4)	49 (7.7)
*5-years TVR*	145 (6.8)	42 (4.7)	103 (8.4)	50 (4.7)	13 (2.7)	37 (6.2)	95 (9.1)	29 (7.1)	66 (10.4)
**Stent thrombosis**									
*5-years ST*	7 (0.3)	2 (0.2)	5 (0.4)	4 (0.4)	1 (0.2)	3 (0.5)	4 (0.4)	1 (0.2)	3 (0.5)

**All-cause death**									
*30 days all-cause death*	12 (0.6)	7 (0.8)	5 (0.4)	5 (0.5)	2 (0.4)	3 (0.5)	5 (0.5)	2 (0.4)	3 (0.5)
*1-year all-cause death*	75 (3.5)	36 (4.1)	39 (3.2)	30 (2.8)	13 (2.7)	17 (2.9)	30 (2.9)	13 (2.7)	17 (2.9)
*5-years all-cause death*	152 (7.2)	68 (7.7)	84 (6.8)	63 (5.9)	27 (5.7)	36 (6.1)	63 (6.0)	27 (5.7)	36 (6.1)

Values are presented as absolute number n (%), MACE includes TLR, TVR, ST and All-cause death, BP-DES: biodegradable polymer drug eluting stent, DP-DES: durable polymer drug eluting stent, MACE: Major Adverse Cardiac Events, TLR: Target Lesion Revascularization, TVR: Target Vessel Revascularization, ST: Stent Thrombosis.

**Fig. 5:**
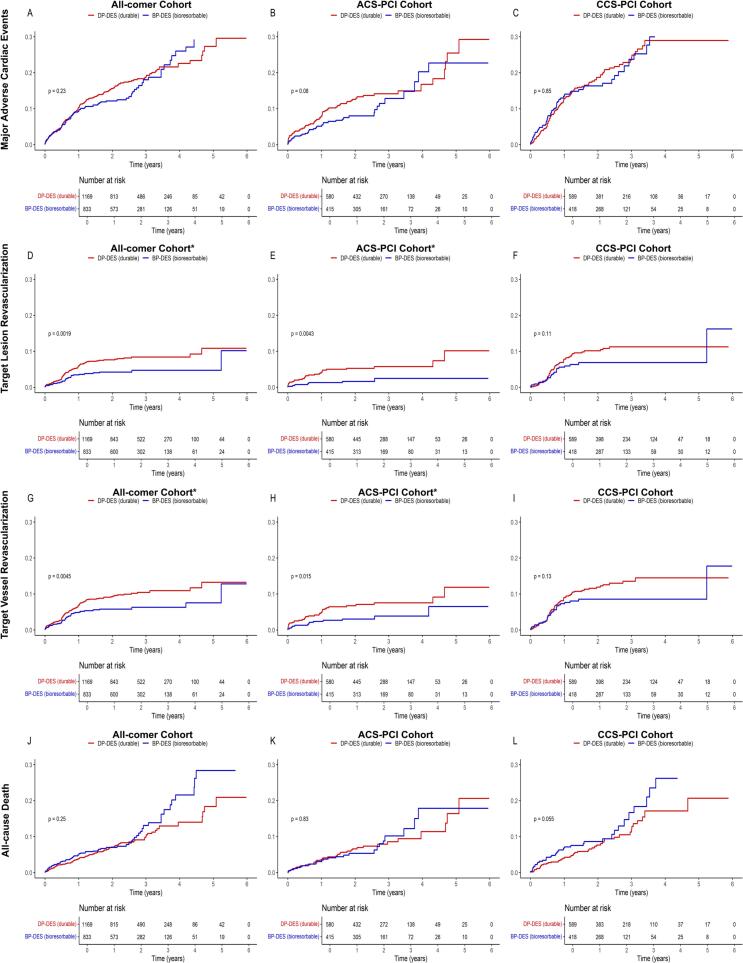
IPW-adjusted Kaplan-Meier curves for clinical outcomes in BP-DES vs. DP-DES.

MACE occurred in 106 patients treated with BP-DES (12%) compared to 179 patients treated with DP-DES (14.5%). The landmark analysis for the cumulative incidence of MACE at the 5-years follow-up revealed no significant difference between both groups (log-rank p: 0.35). Similarly, multivariate Cox regression analysis identified no significant advantage for the use of BP-DES at the 5-years MACE follow-up (OR 0.941, 95% CI: 0.734–1.207, p-value: 0.631; Table 3). IPW-weighted analysis further strengthened these findings (OR 0.91, 95% CI: 0.70–1.17, p-value: 0.451; Table 5, Fig. 5).

### 3.3 Target lesion revascularization

**Fig. 4:**
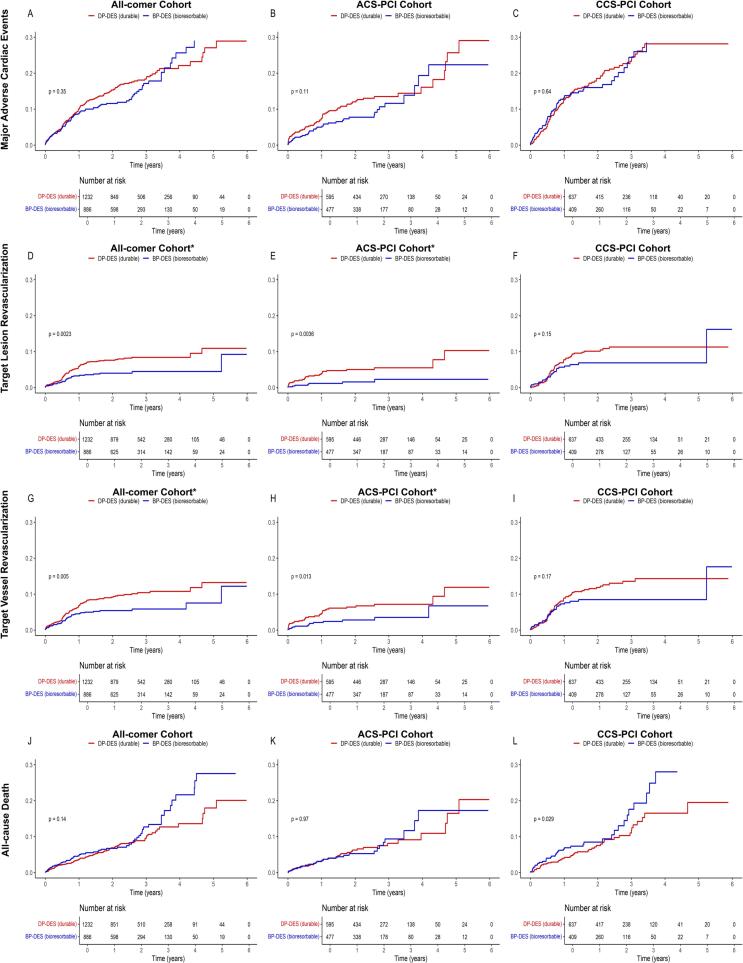
Kaplan-Meier curves for clinical outcomes in BP-DES vs. DP-DES.

Clinically driven target lesion revascularization occurred in 30 patients treated with BP-DES (3.4%) compared to 84 patients in the DP-DES group (6.8%). The landmark analysis at the 5-years follow-up provided a statistically significant reduction for TLR rates favouring BP-DES (OR 0.567, 95% CI: 0.372–0.865, p-value: 0.008). The Kaplan Meier curve and log-rank test supported this result, showing a significant difference between both treatment groups in the all-comer cohort favouring BP-DES, as seen in Fig. 4 (log rank p: 0.002). The results from the IPW-weighted cohort supported these results (OR 0.57, 95% CI: 0.37–0.88, p-value: 0.011; Table 5, Fig. 5).

### 3.4 Subgroup ACS (n = 1072) and CCS PCI cohort (n = 1046)

The median age of patients in the ACS-PCI cohort was 61 years (ICR: 53–72), which was 9 years younger than the CCS-PCI cohort (70 years, ICR: 61–78). The distribution of sex (Male Sex: 73.9% vs 72.7%) and BMI (27.0 vs 27.2) was comparable between the ACS and CCS cohorts. Hypertension was more frequent in ACS patients (92.8% vs 86.8%), while diabetes mellitus (25.0% vs 34.6%) was less frequently registered among ACS PCI patients.

Regarding the interventional characteristics, culprit lesion, median stent length and the median maximum stent diameter were similar. As expected, the median troponin t levels (127.5 vs 27.0) as well as the CK-MB level (64.6 vs 20.7) were lower in CCS patients.

Within the ACS-PCI cohort, clinically driven target lesion revascularization occurred in 7 patients treated with BP-DES (1.5%) compared to 29 patients treated with DP-DES (4.9%). The 5-years landmark analysis revealed a statistically significant benefit for BP-DES usage in patients with ACS (OR 0.364 95% CI: 0.158–0.841, p-value: 0.018). The Kaplan Meier estimate for the cumulative incidence of TLR (Fig. 3) supports the favourable outcome for BP-DES usage (log-rank p-value: 0.004).

In contrast, no significant differences were observed between BP-DES and DP-DES in patients with chronic coronary syndrome (Table 3).

## 4 Discussion

The mechanisms underlying restenosis following the implantation of a DES are complex, multifactorial, and not fully understood. While advances in intracoronary imaging have enabled the optimization of device-related factors, such as stent malposition or underexpansion, restenosis itself continues to be a significant clinical issue. It is increasingly clear that post-PCI vessel injury triggers an inflammatory cascade involving fibroblasts and smooth muscle cells, which contribute to neointimal hyperplasia and progressive vessel re-narrowing [9], [10], [11], [12].

Beyond the antiproliferative effects of DES polymers, future post-PCI therapeutic strategies might focus on anti-inflammatory agents. For example, thiazolidinediones have been associated with reduced TLR rates, suggesting a protective effect potentially mediated through PPAR-γ activation [13]. Other well-known risk factors for TLR include diabetes mellitus, previous PCI, high CRP levels, small stent diameters or an overall higher total stent length [14], [15].

Early generation DP-DES were known to cause suboptimal reendothelialization of the stented region compared to BMS [16]. However, newer generation DP-DES feature thinner stent struts, updated antiproliferative agents and refined polymer coatings, resulting in reduced thrombogenicity and improved vascular healing [2], [17].

Whether BP-DES offer superior patient safety and clinical outcomes compared to DP-DES is part of an ongoing debate, although observed differences are generally marginal [4], [5], [6].

Over the past decades, multiple studies have reported various results. Early trials involving first generation DP-DES often demonstrated superior outcomes for BP-DES in terms of target lesion revascularization and stent thrombosis rates [17], [18], [19]. More recent studies revealed similar outcome rates for both technologies, largely attributed to the development of newer generation DP-DES incorporating improved biocompatibility properties. Some studies suggest a favourable outcome for BP-DES in patients treated for ACS, with modest reductions of TLR, TVR and ST rates, although no significant differences have been observed in cardiac-death rates [20], [21], [22], [23], [24], [25], [26].

Pivotal studies comparing BP-DES vs DP-DES include BIOSCIENCE, BIOFLOW V and BIOSTEMI. BIOSCIENCE demonstrated non-inferiority of BP-DES compared with DP-DES at long-term 5-year follow-up [20]. The following BIOFLOW V study continued to report positive results for BP-DES, as superiority of BP-DES compared to DP-DES was demonstrated at a shorter term 12-month follow-up [5]. However, neither trial was able to demonstrate a clear long-term clinical benefit of BP-DES over DP-DES. More recent data from the BIOSTEMI 5-year follow-up trial might renew the long-standing discussion of whether BP-DES are truly superior to DP-DES, as this study was the first randomized trial to demonstrate an advantage of BP-DES regarding target lesion failure rates at 5-year long-term follow-up [27].

In our study, long-term follow-up data did not demonstrate a significant overall difference between BP-DES and DP-DES, regardless of the clinical indication. Among patients with ACS as the primary indication, MACE occurred less often in patients treated with BP-DES, compared to DP-DES treated patients. These results suggest a modest, but statistically non-significant advantage for BP-DES. These findings are consistent with multiple randomized controlled trials (RCTs) that have compared BP-DES and DP-DES in ACS populations, with a significant risk reduction for the composite endpoint of target lesion failure (TLF), analogous to the MACE findings in this study [25], [27], [28].

Similarly, multiple studies have reported comparable outcomes concerning MACE or TLF in all-comer populations [29], [30], [31]. In our study, the incidence of MACE within the CCS PCI subgroup was similarly distributed between BP-DES and DP-DES treated patients.

### 4.1 MACE

Consequently, while patients with ACS undergoing PCI might benefit from BP-DES treatment, the use of BP-DES in CCS PCI patients did not demonstrate a significant improvement in MACE rates. In the adjusted multivariate regression models, neither subgroup showed a statistically significant difference for the use of BP-DES (Table 4, Table 5).

**Table 4:** Adjusted Multivariate Cox Regression Model for the primary and secondary outcome measures at 5-years All-comer-, ACS- and CCS-PCI Cohort, unweighted cohort.

	**Major Adverse Cardiac Events**		**Target Lesion Revascularization**		**Target Vessel Revascularization**		**Stent Thrombosis**	
	**OR 95% CI**	**p-value**	**OR 95% CI**	**p-value**	**OR 95% CI**	**p-value**	**OR 95% CI**	**p-value**
**All-Comer Cohort**								
**Bioresorbable Polymer**	0.94 (0.73-1.21)	0.631	0.57 (0.37-0.87)	**0.008**	0.61 (0.42-0.88)	**0.009**	0.54 (0.10-2.79)	0.458
**Age, years**	1.02 (1.01-1.03)	**0.002**	0.98 (0.96-0.99)	**0.037**	0.98 (0.96-1.00)	**0.016**	0.96 (0.89-1.04)	0.313
**Male sex**	1.28 (0.97-1.70)	0.086	1.13 (0.71-1.80)	0.605	1.21 (0.79-1.86)	0.378	1.36 (0.16-11.65)	0.778
**Obesity**	1.25 (0.95-1.63)	0.106	0.99 (0.66-1.51)	0.976	1.20 (0.83-1.73)	0.327	1.02 (0.19-5.35)	0.986
**Diabetes**	1.27 (0.99-1.63)	0.061	1.67 (1.14-2.46)	**0.009**	1.42 (1.00-2.03)	0.051	0.48 (0.06-4.12)	0.500
**eGFR, ml/min**	0.99 (0.98-0.99)	**0.001**	0.99 (0.99-1.01)	0.598	1.00 (0.99-1.00)	0.203	1.02 (0.97-1.07)	0.431
**CRP, mg/dl**	1.04 (1.01-1.08)	**0.005**	1.03 (0.98-1.09)	0.222	1.02 (0.96-1.07)	0.567	0.66 (0.20-2.17)	0.494
**PCI Indication: ACS**	0.70 (0.54-0.91)	**0.006**	0.37 (0.24-0.57)	**0.001**	0.45 (0.31-0.66)	**0.001**	0.64 (0.13-3.23)	0.591

**ACS-PCI Cohort**								
**Bioresorbable Polymer**	0.81 (0.54-1.21)	0.295	0.36 (0.16-0.84)	**0.018**	0.48 (0.25-0.92)	**0.028**	0.41 (0.04-3.95)	0.437
**Age, years**	1.05 (1.03-1.07)	**0.001**	1.00 (0.97-1.04)	0.978	1.00 (0.97-1.03)	0.836	0.95 (0.85-1.06)	0.342
**Male sex**	1.65 (1.04-2.63)	**0.033**	1.60 (0.64-3.980)	0.311	1.48 (0.70-3.14)	0.306	0.48 (0.05-4.95)	0.536
**Obesity**	1.57 (1.01-2.45)	**0.046**	1.25 (0.58-2.68)	0.574	1.54 (0.82-2.88)	0.178	2.87 (0.38-21.43)	0.304
**Diabetes**	1.47 (0.98-2.22)	0.062	1.04 (0.49-2.24)	0.916	1.07 (0.56-2.04)	0.848	0.000 (0.00-inf)	0.977
**eGFR, ml/min**	1.00 (0.99-1.01)	0.464	1.00 (0.98-1.02)	0.950	1.00 (0.98-1.02)	0.941	1.01 (0.94-1.08)	0.835
**CRP, mg/dl**	1.08 (1.04-1.12)	**0.001**	1.09 (1.03-1.16)	**0.002**	1.07 (1.01-1.14)	**0.016**	0.33 (0.02-6.94)	0.474

**CCS-PCI Cohort**								
**Bioresorbable Polymer**	1.06 (0.77-1.45)	0.730	0.68 (0.41-1.11)	0.124	0.69 (0.44-1.10)	0.121	0.77 (0.07-8.62)	0.831
**Age, years**	1.00 (0.99-1.02)	0.603	0.97 (0.95-0.99)	**0.014**	0.97 (0.95-0.99)	**0.002**	0.97 (0.86-1.09)	0.652
**Male sex**	1.13 (0.79-1.61)	0.494	0.99 (0.58-1.70)	0.965	1.10 (0.65-1.85)	0.723	-	-
**Obesity**	1.14 (0.81-1.59)	0.456	0.94 (0.57-1.55)	0.812	1.11 (0.70-1.74)	0.663	-	-
**Diabetes**	1.11 (0.81-1.52)	0.524	1.88 (1.18-2.98)	**0.007**	1.52 (0.98-2.33)	0.059	1.25 (0.11-14.00)	0.856
**eGFR, ml/min**	0.98 (0.98-0.99)	**0.001**	1.00 (0.99-1.01)	0.574	0.99 (0.99-1.00)	0.157	1.03 (0.96-1.11)	0.433
**CRP, mg/dl**	1.01 (0.96-1.06)	0.773	0.92 (0.79-1.07)	0.275	0.90 (0.77-1.05)	0.175	0.87 (0.32-2.36)	0.789

OR: Adjusted Odds Ratio, eGFR: estimated glomerular filtration rate, CRP: c-reactive protein, PCI: Percutaneous Coronary Intervention, ACS: Acute Coronary Syndrome, eGFR: estimated glomerular filtration rate, CRP: c-reactive protein

### 4.2 TLR

Regarding the secondary endpoint in both the ACS PCI and the CCS PCI cohort, TLR was less frequently observed in patients treated with BP-DES compared to those treated with DP-DES during the 5-years follow-up period (Table 3). In CCS PCI patients, no significant result was obtained regarding the usage of BP-DES for clinically driven TLR (Table 4, Table 5). However, ACS-PCI patients treated with BP-DES demonstrated a statistically significant risk decrease for TLR compared to patients treated with DP-DES, which is thought to mainly contribute to the overall lower rate of MACE in the all-comer population patients treated with BP-DES. These findings align with previously reported study outcomes [25], [27], [28], [32]. Although the observed reduction in TLR among patients with ACS was statistically significant, the absolute risk reduction was modest, and event numbers were small. Therefore, these findings should be interpreted with caution, as they may reflect operator behaviour or lesion selection rather than stent-related biological effects. A possible explanation of the difference between DP-DES and BP-DES regarding this endpoint may be the distinct pathophysiology of the treated lesions. Myocardial infarction (MI) lesions are often characterized by thin cap fibroatheromas (TCFA), larger necrotic cores, and stronger inflammatory activity. These features may lead to delayed and altered endothelialization after stent implantation compared with the more stable lesions seen in CCS [33], [34]. Additionally, MI-lesions have been shown to exhibit slower endothelialization than CCS lesions [35]. Therefore, a permanent pro-inflammatory polymer may further promote neoatherosclerosis and contribute to higher rates of TLR, whereas patients may benefit from BP-DES usage in this regard. Importantly, our data did not show any effect on all-cause mortality.

**Table 5:** Adjusted Multivariate Cox Regression Model for the primary and secondary outcome measures at 5-years All-comer-, ACS- and CCS-PCI Cohort (IPW adjusted).

	**Major Adverse Cardiac Events**		**Target Lesion Revascularization**		**Target Vessel Revascularization**	
	**OR 95% CI**	**p-value**	**OR 95% CI**	**p-value**	**OR 95% CI**	**p-value**
**All-comer Cohort**						
**Bioresorbable Polymer**	0.91 (0.70–1.17)	0.451	0.57 (0.37–0.88)	**0.011**	0.61 (0.42–0.90)	**0.012**
**Age, years**	1.02 (1.01–1.03)	**0.004**	0.98 (0.96–1.00)	**0.028**	0.98 (0.96–0.99)	**0.009**
**Male sex**	1.20 (0.90–1.60)	0.209	1.04 (0.65–1.65)	0.885	1.11 (0.72–1.71)	0.622
**Obesity**	1.23 (0.94–1.62)	0.131	1.01 (0.67–1.55)	0.947	1.22 (0.84–1.77)	0.298
**Diabetes**	1.27 (0.98–1.64)	0.067	1.61 (1.10–2.38)	**0.016**	1.38 (0.97–1.96)	0.077
**eGFR, ml/min**	0.99 (0.98–0.99)	**<0.001**	1.00 (0.99–1.01)	0.650	1.00 (0.99–1.00)	0.225
**CRP, mg/dl**	1.05 (1.01–1.08)	**0.012**	1.04 (0.97–1.11)	0.319	1.02 (0.95–1.09)	0.642
**PCI Indication: ACS**	0.73 (0.55–0.95)	**0.019**	0.38 (0.25–0.59)	**<0.001**	0.47 (0.32–0.70)	**<0.001**

**ACS-PCI Cohort**						
**Bioresorbable Polymer**	0.80 (0.53–1.20)	0.272	0.38 (0.16–0.90)	**0.027**	0.50 (0.25–0.98)	**0.042**
**Age, years**	1.05 (1.02–1.07)	**<0.001**	1.00 (0.96–1.04)	0.899	1.00 (0.97–1.03)	0.958
**Male sex**	1.54 (0.98–2.41)	0.059	1.53 (0.63–3.70)	0.343	1.39 (0.67–2.89)	0.373
**Obesity**	1.50 (0.94–2.38)	**0.086**	1.17 (0.49–2.79)	0.728	1.43 (0.71–2.86)	0.314
**Diabetes**	1.54 (1.03–2.31)	**0.036**	1.11 (0.48–2.57)	0.812	1.11 (0.57–2.18)	0.760
**eGFR, ml/min**	1.00 (0.99–1.01)	0.394	1.00 (0.98–1.02)	0.963	1.00 (0.99–1.01)	0.983
**CRP, mg/dl**	1.07 (1.03–1.12)	**0.001**	1.08 (1.01–1.17)	**0.034**	1.07 (0.99–1.15)	0.097

**CCS-PCI Cohort**						
**Bioresorbable Polymer**	1.01 (0.73–1.40)	0.962	0.68 (0.41–1.12)	0.132	0.69 (0.43–1.11)	0.126
**Age, years**	1.00 (0.99–1.02)	0.584	0.97 (0.95–0.99)	**0.005**	0.97 (0.95–0.99)	**<0.001**
**Male sex**	1.06 (0.74–1.53)	0.756	0.88 (0.50–1.54)	0.651	0.99 (0.58–1.70)	0.976
**Obesity**	1.15 (0.83–1.60)	0.427	0.98 (0.60–1.60)	0.939	1.16 (0.74–1.81)	0.513
**Diabetes**	1.08 (0.78–1.49)	0.629	1.75 (1.11–2.78)	**0.017**	1.42 (0.92–2.19)	0.110
**eGFR, ml/min**	0.99 (0.98–0.99)	**<0.001**	1.00 (0.99–1.01)	0.674	0.99 (0.99–1.00)	0.199
**CRP, mg/dl**	1.01 (0.97–1.07)	0.582	0.91 (0.76–1.10)	0.336	0.89 (0.73–1.08)	0.245

OR: Adjusted Odds Ratio, eGFR: estimated glomerular filtration rate, CRP: c-reactive protein, PCI: Percutaneous Coronary Intervention, ACS: Acute Coronary Syndrome, eGFR: estimated glomerular filtration rate, CRP: c-reactive protein

The implications of these results remain uncertain, prompting questions about whether optimizing other procedural factors, such as patient related considerations, might offer greater benefits in an already very safe procedure [36].

### 4.3 Strengths and limitations

This study effectively included a large patient cohort for a comparably long follow-up time. Due to the characteristics of this study design, the analysis is susceptible to various biases. Treatment choice for either BP-DES or DP-DES PCI was not randomized and distributed proportionately but based on current hospital practice and physician preference. Different stent platforms were pooled, which may have introduced residual confounding due to heterogeneity in stent-specific characteristics. Stent thrombosis rates were low and underreporting cannot be excluded due to the nature of registry-based outcome collection. Furthermore, detailed periinterventional and lesion specific data, such as physiological lesion assessment (e.g., FFR), intravascular imaging (e.g., IVUS) and detailed lesion complexity variables (e.g., SYNTAX score) were not available from our database. As these factors may influence restenosis rates, residual confounding cannot be fully excluded.

## 5 Conclusion

Biodegradable polymer drug-eluting stents and durable polymer drug-eluting stents demonstrate comparable safety profiles regarding the composite endpoint MACE at the 5-years long-term follow-up. BP-DES were associated with lower rates of the secondary individual endpoint clinically driven TLR in patients undergoing primary PCI for ACS.

## Author contributions

C.S. and J.R. contributed to data collection, drafting of the manuscript, and critical revision. A.M.D., G.M.S.G., M.P., C.E., A.T., W.S.S., N.S.S., C.G., I.M.L. and J.M.S.M contributed to the critical revision and proofreading of the manuscript. All authors reviewed and approved the final version of the manuscript.

## CRediT authorship contribution statement

**Christof Skos:** Writing – review & editing, Writing – original draft, Visualization, Validation, Software, Resources, Project administration, Methodology, Investigation, Formal analysis, Data curation, Conceptualization. **Jovan Rogozarski:** Writing – review & editing, Software, Project administration, Data curation. **Al Medina Dizdarevic:** Writing – review & editing, Project administration, Investigation, Data curation. **Gloria M.Steiner-Gager:** Writing – review & editing, Supervision, Investigation. **Marek Postula:** Writing – review & editing. **Ceren Eyileten:** Writing – review & editing. **Aurel Toma:** Writing – review & editing. **Walter S. Speidl:** Writing – review & editing. **Nika Skoro-Sajer:** Writing – review & editing. **Christian Gerges:** Writing – review & editing. **Irene M. Lang:** Writing – review & editing. **Jolanta M. Siller-Matula:** Writing – review & editing, Visualization, Validation, Supervision, Project administration, Methodology, Investigation, Formal analysis, Data curation, Conceptualization.

## Declaration of competing interest

The authors declare that they have no known competing financial interests or personal relationships that could have appeared to influence the work reported in this paper.
