# Supplementary material for: Outcomes of durable versus biodegradable polymer drug-eluting stents in patients with coronary artery disease
Source: Int J Cardiol Heart Vasc. 2026 Feb 6;63:101882. doi: 10.1016/j.ijcha.2026.101882 (PMC12906131; doi:10.1016/j.ijcha.2026.101882)
Supplement: Supplementary Data 1 [file mmc1.docx]

| **Supplementary Table S1** Baseline Characteristics All Comer Cohort after IPW Calculation | | | | |
| --- | --- | --- | --- | --- |
|  |  | | |  |
|  | **All-comer cohort (n = 2005)** | | |  |
|  | **Overall  *(n=2005)*** | **BP-DES *(n=837)*** | **DP-DES *(n=1168)*** | ***p-value*** |
| Age (years) | 66 (56-76) | 67 (56-76) | 65 (57-76) | 0.672 |
| Male Sex | 1476 (73.6) | 617 (73.7) | 859 (73.5) | 0.932 |
| **Risk Factors / Comorbidities, n (%)** |  |  |  |  |
| Body mass index, kg/m^2^ | 27.1 (24.7-30.6) | 27.1 (24.7-30.4) | 27.1 (24.6-30.8) | 0.563 |
| Obesity, n (%) | 542 (27.0) | 214 (25.6) | 328 (28.1) | 0.217 |
| Hypertension | 1796 (89.6) | 748 (89.7) | 1048 (89.4) | 0.794 |
| Dyslipidaemia Treatment | 2005 (100.0) | 837 (100.0) | 1168 (100.0) | . |
| Diabetes mellitus | 602 (30.0) | 250 (29.9) | 352 (30.1) | 0.911 |
| **Interventional Characteristics** |  |  |  |  |
| Elective PCI | 1010 (50.4) | 422 (50.5) | 588 (50.3) | 0.952 |
| STEMI | 607 (30.3) | 256 (30.6) | 351 (30.1) | 0.784 |
| NSTEMI | 387 (19.3) | 158 (18.9) | 229 (19.6) | 0.683 |
| Culprit lesion: LAD | 958 (47.8) | 399 (47.7) | 559 (47.9) | 0.933 |
| Culprit lesion: RCA | 554 (27.6) | 232 (27.8) | 322 (27.6) | 0.928 |
| Culprit lesion: RCX | 357 (17.8) | 149 (17.8) | 208 (17.8) | 0.997 |
| Culprit Lesion: LM | 22 (1.1) | 9 (1.1) | 13 (1.1) | 0.937 |
| Total number of stents (IQR) | 1.0 (1.0-2.0) | 1.0 (1.0-2.0) | 1.0 (1.0-2.0) | 0.654 |
| Total stent length, mm | 27.0 (18.0-41.0) | 27.0 (18.0-40.0) | 28.0 (18.0-41.0) | 0.075 |
| Maximum diameter stent, mm | 3.0 (3.0-4.0) | 3.0 (3.0-4.0) | 3.0 (3.0-4.0) | **0.034** |
| Fluoroscopy time, minutes | 16 (10-26) | 17 (10-26) | 16 (10-26) | 0.274 |
| Contrast volume, ml | 180 (130-250) | 181 (130-250) | 180 (132-250) | 0.097 |
| Dose Area Product (DAP), cGycm^2^ | 7460 (4353-13043) | 6984 (3970-12217) | 7823 (4527-14001) | **0.012** |
| **Laboratory Data** |  |  |  |  |
| Troponin T, pg/ml | 61 (22-298) | 53 (21-227) | 65 (22-337) | 0.360 |
| NTproBNP, pg/ml | 410.3 (113.5-1530.0) | 389.8 (108.2-1496.0) | 427.4 (116.9-1570.0) | 0.306 |
| CK, U/L | 121 (73-262) | 108 (70-226) | 128 (78-284) | **0.001** |
| CK-MB, U/L | 45.2 (22.1-111.7) | 47.3 (21.8-120.7) | 42.9 (22.1-107.8) | **0.036** |
| Haemoglobin, g/dl | 13.9 (12.5-15.1) | 13.8 (12.4-15.1) | 13.9 (12.6-15.1) | 0.518 |
| HbA1c, % (mmol/mol) | 5.7 (5.4-6.2) | 5.7 (5.4-6.2) | 5.7 (5.4-6.3) | 0.085 |
| eGFR, ml/min | 78.2 (58.1-92.6) | 77.6 (57.9-92.4) | 78.5 (58.4-92.8) | 0.254 |
| LDL-cholesterol, mg/dl | 92.4 (62.8-122.2) | 92.4 (62.4-123.2) | 92.4 (63.2-119.8) | 0.438 |
| Triglycerides, mg/dl | 123 (85-176) | 124 (89-179) | 122 (84-175) | 0.253 |
| Leukocytes, /l | 9.1 (7-11.7) | 9.0 (7.0-11.6) | 9.1 (7.0-11.7) | 0.833 |
| CRP, mg/dl | 0.4 (0.2-1.0) | 0.3 (0.1-1.0) | 0.4 (0.2-0.9) | 0.119 |
| **Concomitant Medication** |  |  |  |  |
| Aspirin | 1783 (88.9) | 750 (89.6) | 1033 (88.4) | 0.413 |
| DOAC | 230 (11.5) | 107 (12.8) | 123 (10.5) | 0.120 |
| P2Y inhibitor | 1631 (81.3) | 687 (82.1) | 944 (80.8) | 0.442 |
| Beta-blocker | 1678 (83.7) | 703 (84.0) | 975 (83.5) | 0.758 |
| Calcium channel blocker | 404 (20.1) | 162 (19.4) | 242 (20.7) | 0.461 |
| ACE inhibitor | 1130 (56.4) | 490 (58.6) | 640 (54.8) | 0.089 |
| ARB | 624 (31.1) | 241 (28.8) | 383 (32.8) | 0.059 |
| Statin | 1976 (98.6) | 826 (98.8) | 1150 (98.5) | 0.618 |
|  |  |  |  |  |
| **DOAC:** direct oral anticoagulant; **NTproBNP:** N-terminal pro b-type natriuretic peptide; **LDL:** low-density lipoprotein, **HbA1c:** glycated haemoglobin; **eGFR:** estimated glomerular filtration rate, **CRP:** c-reactive protein, **PCI:** percutaneous coronary intervention, **DES:** drug-eluting stent, **CK:** creatine kinase, **Lp-PLA2:** lipoprotein-associated phospholipase A2, **ACE:** angiotensin-converting enzyme inhibitor, **ARB:** angiotensin receptor blocker, **DAP:** dose area product, (**N)IDDM:** (non-)insulin dependent diabetes, **(N)STEMI:** (non-)ST-elevation myocardial infarction, **LAD:** left anterior descending artery, **RCA:** right coronary artery, **RCX:** ramus circumflex artery, **LM:** left main coronary artery | | | | |
|  |  |  |  |  |
